# Effects of previous orthodontic treatment on periodontal status of patients in long-term supportive periodontal care

**DOI:** 10.1186/s12903-025-07534-6

**Published:** 2025-12-17

**Authors:** Sarah K. Sonnenschein, Alexander-Nicolaus Spies, Christopher Büsch, Sinclair Awounvo, Sinan Şen, Ti-Sun Kim

**Affiliations:** 1 Department of Conservative Dentistry, Clinic for Oral-, Dental- and Maxillofacial Diseases, Heidelberg University, Heidelberg University Hospital, Im Neuenheimer Feld 400, Heidelberg, 69120 Germany; 2 Institute of Medical Biometry, Heidelberg University, Heidelberg, Germany; 3 Department of Orthodontics and Dentofacial Orthopaedics, Clinic for Oral-, Dental- and Maxillofacial Diseases, Heidelberg University, Heidelberg University Hospital, Heidelberg, Germany; 4 Department of Orthodontics, University Hospital Schleswig-Holstein, Campus Kiel, Kiel, Germany

**Keywords:** Supportive periodontal care, Orthodontics, Malocclusion, Three-Dimensional images, Dental aesthetics

## Abstract

**Background:**

While many German children and young adults receive orthodontic treatment (OTx), the number of patients requiring periodontal treatment is increasing due to demographic changes. Investigating the long-term effects of orthodontic treatment on periodontal health, particularly in patients developing periodontitis, is therefore of public health interest. Primary aim was to evaluate whether an anamnestic history of OTx affects the progression of periodontal parameters over a ten-year period of supportive periodontal care (SPC). Additionally, the study aimed to determine whether orthodontic treatment need in SPC patients correlates with periodontal and dental parameters change during the preceding ten years of SPC.

**Methods:**

Sixty periodontitis patients with ten years (± six months) of SPC received digital intraoral scans during cross-sectional SPC follow-up examination (T1). Patients’ previous orthodontic treatment (POT) or no treatment (NOT) was recorded. The Index of Orthodontic Treatment Need (IOTN) at T1 was assessed. Dental and periodontal parameters were recorded and compared with retrospective data from ten years (± six months) earlier (T0). The association between changes in clinical attachment levels (CAL T0-T1) and treatment group (POT/NOT) was analysed (multiple linear regression). Spearman correlation between IOTN and clinical parameters change was assessed.

**Results:**

The change in parameters from T0 to T1 was as follows (POT: *n* = 24 patients, NOT: *n* = 36 patients): Mean tooth loss: 0.92 ± 1.74 vs. 0.64 ± 0.90; Mean probing pocket depth: -0.03 ± 0.33 mm vs. 0.05 ± 0.51 mm; Mean CAL: 0.11 ± 0.59 mm vs. 0.09 ± 0.66 mm. No association was found between CAL change and treatment group. Only a negligible correlation between IOTN and changes in dental, periodontal, and oral hygiene parameters was found.

**Conclusions:**

Patients with successfully treated periodontitis, both with and without a history of orthodontic treatment, show a high level of periodontal stability during long-term SPC and comparable orthodontic conditions.

**Trial registration:**

Clinical trial registration number on the German clinical trials register: DRKS00011316 (Registration date 17th November 2016).

## Introduction

Periodontitis is a chronic, plaque-induced and multifactorial inflammatory disease of the periodontium., The global prevalence of severe periodontitis is 9.8% [1]. However, prevalence varies between different countries and age groups. In the most recent German Oral Health Study, a community periodontal index score of 4, which indicates severe periodontitis, occurred in 42.4% of younger seniors (age 65 to 74) [2]. Systematic periodontitis therapy is divided into active periodontitis therapy (APT) and supportive periodontal care (SPC), which begins once stable conditions have been reached. Lifelong SPC is necessary to prevent a recurrent progression of periodontitis or to enable an immediate re-instrumentation in case of recurrence, which manifests itself in gingival inflammation, increased probing pocket depth (PPD) or bleeding gums [3]. SPC includes periodontal health monitoring, oral hygiene instructions, professional mechanical biofilm removal, localised subgingival instrumentation of residual pockets and patient-specific behavioural instructions [4]. Overall, high adherence with SPC seems to have a positive impact on the periodontal situation [5].

Tooth misalignments can have a negative effect on the functionality and lead to malocclusion. Certain tooth malocclusions make oral hygiene more difficult, which makes it harder to clean the teeth and can have a negative effect on the periodontal situation [6, 7]. Tooth misalignments manifest primarily in adolescence and are treated most efficiently at this time due to the physiological conditions. Uncontrolled and excessive orthodontic forces however can lead to the destruction of the periodontium [8, 9]. Since not every malocclusion requires treatment, several indices have been developed to classify the need for orthodontic treatment (OT) [10]. The Index of Orthodontic Treatment Need (IOTN) was developed by Brook & Shaw in 1989 [11] and consists of the dental health component (DHC) and an aesthetic component (AC). The IOTN can be determined digitally using three-dimensional digital models (3DM) that were assessed by digital intraoral scanning (IOS). IOS is commonly used in various areas of dentistry [12]. Studies have shown that especially in patients with a periodontally compromised dentition, digital impression taking using IOS is superior to conventional methods [13, 14]. Consequently, assessing the IOTN in these patients using 3DMs from IOS represents a suitable alternative.

Various studies exist on the long-term impact of OTx and malocclusions on the periodontium [15–20]. A report of the Institute for Health and Social Research concluded, that the few existing studies on long-term effects of OTx on oral health are only suitable for approximating the consequences of OTx [21]. In two recently published meta-analyses, low quality and a large variation of the included studies were reported [22, 23]. Few studies examined the orthodontic treatment need in periodontitis patients, but periodontal parameters were only recorded at one point in time [24, 25]. A recent systematic review found that OTx resulted in lower probing depths and clinical attachment gains in systematically treated periodontitis patients. The authors concluded that OTx can be used for patients with reduced periodontal support to stabilise clinical findings and improve function and aesthetics [26]. Roccuzzo et al. observed that even after regenerative periodontitis therapy for severe bone defects, OTx did not reduce the long-term benefits of periodontal regeneration [27]. Overall, there are few studies on the topic of orthodontics and periodontitis. In the future, a large proportion of older periodontitis patients will most likely have undergone orthodontic treatment.

The present exploratory retrospective cohort study aimed to investigate the long-term effects of OTx on periodontal parameters over a ten-year period of SPC. The primary research question of the study was: Does an anamnestic history of OTx affect the progression of periodontal parameters, measured by clinical attachment loss (CAL) and tooth loss due to periodontitis (TLP), over a ten-year period of SPC? Additionally, the study aimed to determine whether orthodontic treatment need in SPC patients correlates with periodontal and dental parameters change during the preceding ten years of SPC.

## Materials & methods

The study was approved by the Institutional Review Board for Human Studies of the Medical Faculty of Heidelberg (#S-132/2014, amendment confirmed: 11th August 2020), is registered in the German Register of Clinical Studies (DRKS00011316, registration date 17th November 2016) and was conducted according to the STROBE guidelines for observational studies [28]. All procedures complied with the ethical standards of the Institutional Research Board and the 1964 Declaration of Helsinki and its subsequent amendments or comparable ethical standards. All participants were informed about the study and the investigations and gave their verbal and written consent to participate. The included patients represent a subpopulation that has been monitored as part of a larger bidirectional cohort study (start of prospective observation: 07/2014–01/2016). Retrospective data of the included patients have been partially published [5, 29, 30]; however, not for the observation time points in the present study and within the scope of different research questions.

### Patient recruitment, and observation time points

Patients with at least 9,5 years of SPC at the authors institution and who had a SPC session between August 2020 and August 2021 were invited to participate. Patients who agreed to participate in the study and met the inclusion criteria, were examined according to the study protocol. This SPC session was defined as the follow-up visit (T1). The SPC session ten years ± six months prior to the respective date of T1 was retrospectively defined as baseline-visit (T0). Inclusion criteria were completed APT and documentation of dental and periodontal status and hygiene indices at both time points.

### Group classification

Patients without orthodontic treatment (NOT) were distinguished from patients who had received and finished active OTx ten years prior to T0 (POT). OTx was defined as the use of fixed or removable orthodontic appliances with or without surgical procedures, including tooth extraction. The extent and duration of OTx were not differentiated for group allocation. Only patients who had received OTx during adolescence (age 10–19 according to the World Health Organization) were included to increase homogeneity of the study population. Patients were excluded from the study if they (i) were < 18 years old, (ii) had < 18 teeth, (iii) had not received APT, (iv) had received active OTx in adulthood or between T0-T1, or if they were (v) diagnosed with periodontitis as a manifestation of systemic diseases.

### Data acquisition

The T1 follow-up examination included an update of the medical history, assessment of the Gingival Bleeding Index (GBI) [31] and the Plaque Control Record (PCR) [32] by experienced, calibrated dental hygienists, followed by professional mechanical biofilm removal. A dental examination including a complete periodontal status with PPD, gingival recessions and CAL at six sites per tooth (periodontal probe PCPUNC15, Hu-Friedy, Chicago, USA), furcation involvement (Nabers probe PQ2N, Hu-Friedy, Chicago, USA) [33], tooth mobility [34] and Bleeding on Probing (BOP) was performed by five calibrated investigators. The periodontal parameters were aggregated for each patient using the average of all values. Due to the similarity of the groups, comparability of assessment methods could be assumed.

Between T0 and T1, the SPC intervals were determined based on the patient’s individual periodontitis risk assessment by Ramseier & Lang (3/6/12 months) [35, 36]. SPC sessions included assessment of oral hygiene indices, re-instruction and professional biofilm removal with manual and motorized mechanical instruments by hygienists. Periodontal and dental status were assessed at least once a year. Pockets with PPD = 4 mm and BOP or PPD ≥ 5 mm were re-instrumented non-surgically with manual instruments and/or Air-Scaler by dentists specialized in periodontology and additional surgical interventions were performed if indicated to achieve a stable periodontal situation [4]. Due to coronavirus restrictions, only hand instruments were used in some T1 sessions to prevent aerosols. Tooth loss (TL) between T0 and T1 was categorised either due to periodontal or due to any other reasons. The primary cause of tooth loss was researched based on patient records, clinical findings, and X-rays. Teeth presenting furcation involvement [33], increased PPD into the apical third of the root at more than one site of the tooth and generalised bone loss around the tooth into the apical third of the root were classified as lost due to periodontal reasons. Tooth hypermobility was also taken into account if the teeth were not splinted. In cases of combined endodontic-periodontal lesions, the primary cause (if identifiable) was evaluated. Retrospective data at T0 included PCR, GBI and anamnestic, dental, and periodontal data. The examinations at T0 were performed by calibrated members of the section of Periodontology at the authors institution. Calibration between investigators at T0 and T1 was performed as a standard procedure at the authors institution for the assessment of the periodontal status. This requires a millimetre-level agreement of more than 90% with a patient reference model. Additionally, the Lin’s concordance correlation coefficient (CCC) indicated a high level of agreement between raters and the patient reference model for both PPD (CCC ≥ 0.89) and CAL (CCC ≥ 0.92). All data at T0 was taken from the respective patient files. Periodontitis stage and grade according to the current classification [37] and clinical endpoints for periodontal trials according to Feres et al. [38] were assessed for T0 and T1 using clinical and radiographic data. Adherence with SPC was assessed according to Sonnenschein et al. [5].

### Intraoral scans and IOTN-assessment

All 3DMs were assessed with Primescan^®^ AC according to a standardised scanning protocol [39], using the Connect Software 5.1.3 (Dentsply Sirona GmbH, Bensheim, Germany). All IOS were performed by two calibrated examiners. The IOTN was determined semi-automatically according to So and Tang [40] using the software OnyxCeph^3TM^ (Dentaurum GmbH & Co. KG, Ispringen, Germany). Orthodontic treatment need at T1 was differentiated into “low”, “borderline” and “high” according to Richmond et al. [41]. Further information on IOTN-assessment and intra-/inter-rater reliability are described in detail in a previous publication [42].

### Statistical analysis

All data was compiled, pseudonymised and digitised by a single examiner. Continuous variables were expressed as mean values ± standard deviation and categorical variables as absolute and relative frequencies. For continuous variables, difference between the independent treatment groups (POT/NOT) at T0, T1, and ΔT0-T1 was assessed using either a Student’s t-test or a Mann-Whitney-U-test after examining the distribution of each variable. Additionally, within-group comparisons between T0 and T1 were performed using either a paired Student’s t-test or a Wilcoxon signed-rank test, depending on the variable distribution. For categorical variables, Pearson’s chi-squared tests were used to compare the distribution of variables across the independent treatment groups (POT/NOT) at T0 and T1. Moreover, within-group comparison of the clinical endpoint attainment between T0 and T1 was conducted using McNemar’s test.

As the endpoint “Mean CAL change between T0 and T1” is continuous, it was analysed using a multiple linear regression. The dependent variable was the difference of the mean CAL between T0 and T1 (in mm), where a value of zero indicates no change in CAL between these two time points. The treatment group (POT/NOT) was included as fixed effect (independent variable) in the model. Additionally, CAL at T0 (in mm), sex (male/female), age at T0 (in years), number of teeth at T0, and smoking status (yes/no) were included as covariates to adjust the estimated treatment group effect. Using the sample size and the observed effect measure of the treatment group regarding CAL change (∆T0-T1), a post-hoc power test for a Student’s t-test was performed.

The secondary endpoint “cumulative incidence of TLP between T0 and T1” was analysed using a negative binomial regression, with the treatment group (POT/NOT) as the fixed effect. Poisson regression was not used due to the presence of over-dispersed data. Additionally, Spearman correlation coefficients were calculated between the IOTN and the change in parameters TL, number of teeth, PPD, BOP, GBI, and PCR between T0 and T1 for all patients regardless of group classification. A p-value < 0.05 was considered statistically significant. The analysis was conducted using R (version 4.2.1, R Core Team, Auckland, New Zealand). Due to the exploratory character of the present study, no sample size calculation was performed and a minimum of 25 patients per group was targeted as a convenience sample.

## Results

### Descriptive statistic of the study cohort

**Fig. 1:**
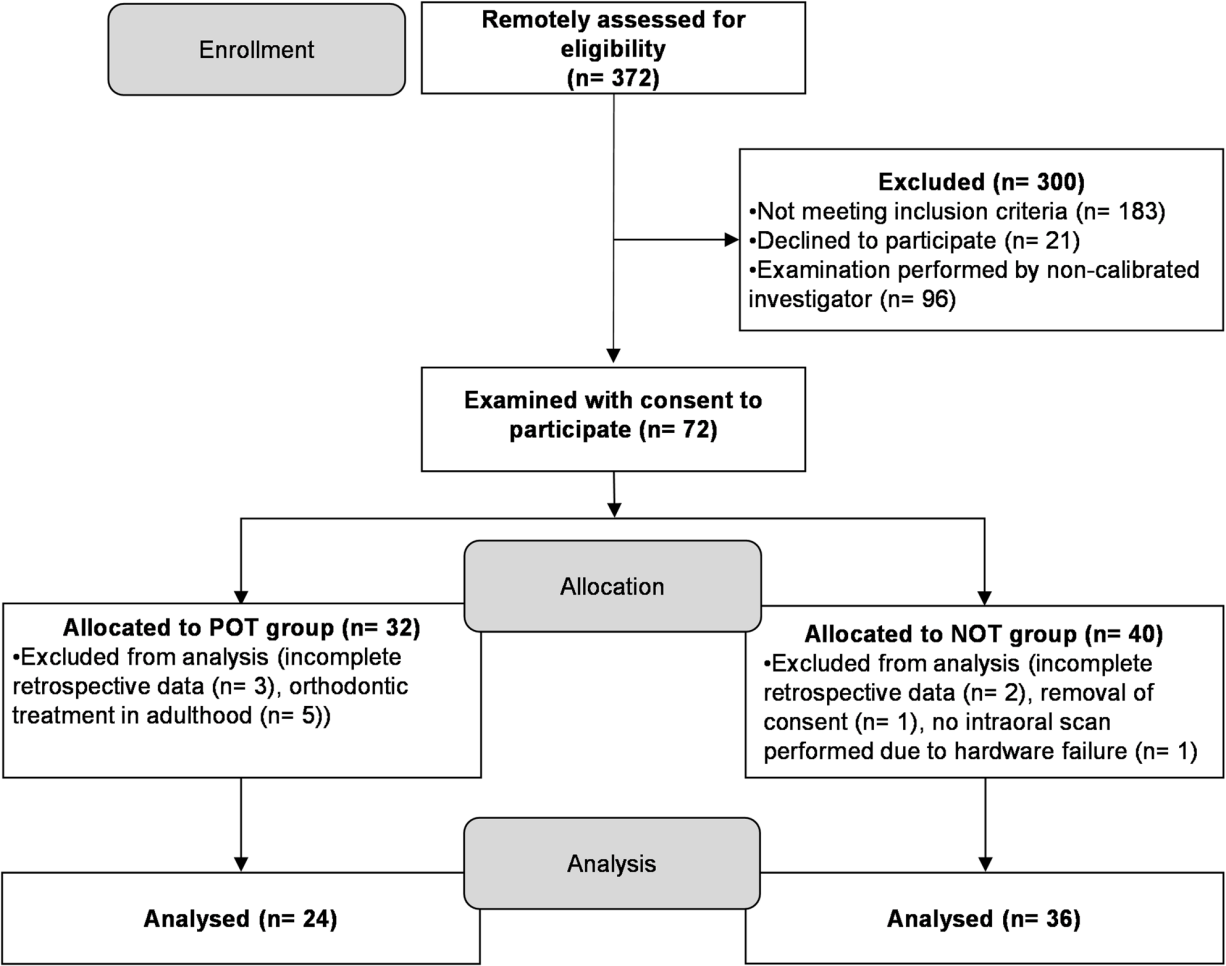
Application of the inclusion and exclusion criteria for the study cohort. n, number of patients; NOT, no orthodontic treatment; POT, previous orthodontic treatment; SPC, supportive periodontal care; T0, retrospective baseline examination ten years (± six months) prior to T1; T1, follow-up examination

Sixty patients were included in the study. The POT-group included 24 patients, the NOT-group 36 patients. A flow diagram including enrolment, allocation and analysis of the study cohort is shown in Fig. 1. Initial assessment for eligibility was performed remotely, screening for patients with at least ten 9,5 years of SPC. Definitive screening for inclusion criteria, informing patients about the study and obtaining their consent was carried out at the appointment. The clinical re-examinations were conducted from August 2020 to July 2021. The individual baselines at T0 ranged from April 2010 to October 2011. The average time period between T0 and T1 was nine years and 11 months (± three months). Table 1 provides a detailed description of the study groups.

**Table 1:** Description of the study groups

*Characteristics*	*POT*		*NOT*			
	*T0*	*T1*	*T0*	*T1*		
Patients [n]	24		36			
Age Mean ± SD (min-max)	51.03 ± 8.96 (35.50–70.75.50.75)	61.07 ± 9.04 (45.33–81.25)	53.28 ± 7.82 (42.17–68.08)	63.18 ± 7.89 (52.00–78.17.00.17)		
Sex, m/w, (%)	12 (50.00%)/12 (50.00%)		24 (66.67%)/12 (33.33%)			
Smoker [n], (%)						
Non-smoker	18 (75.00%)	19 (79.17%)	26 (72.22%)	27 (75.00%)		
Former smoker	3 (12.50%)	3 (12.50%)	3 (8.33%)	4 (11.11%)		
< 10/day	2 (8.33%)	1 (4.17%)	3 (8.33%)	4 (11.11%)		
> 10/day	1 (4.17%)	1 (4.17%)	4 (11.11%)	1 (2.78%)		
Systemic diseases [n], (%)						
Diabetes mellitus	1 (4.17%)	2 (8.33%)	0 (0.00%)	0 (0.00%)		
Other diseases	2 (8.33%)	3 (12.50%)	4 (11.11%)	4 (11.11%)		
Periodontitis stage [n], (%)						
1	0 (0.00%)	0 (0.00%)	0 (0.00%)	0 (0.00%)		
2	2 (8.33%)	1 (4.17%)	4 (11.11%)	1 (2.78%)		
3	20 (83.33%)	21 (87.50%)	30 (83.33%)	32 (88.89%)		
4	2 (8.33%)	2 (8.33%)	2 (5.56%)	3 (8.33%)		
Periodontitis grade [n], (%)						
A	0 (0.00%)	0 (0.00%)	0 (0.00%)	0 (0.00%)		
B	6 (25.00%)	5 (20.83%)	9 (25.00%)	10 (27.78%)		
C	18 (75.00%)	19 (79.17%)	27 (75.00%)	26 (72.22%)		
Visits between T0 and T1						
Number of visits, Mean ± SD (min-max)	18.75 ± 4.75 (11–29)		17.53 ± 3.82 (10–27)			
Annual visits [n], (%)	16 (66.67%)		25 (69,44%)			
One year without visit [n], (%)	6 (25.00%)		8 (22,22%)			
More than one year without visit [n], (%)	2 (8.33%)		3 (8,34%)			
Individual periodontitis risk profile at T0 (according to Ramseier&Lang[35, 36])						
High risk profile [n], (%)	7 (29,17%)		10 (27,78%)			
Moderate risk profile [n], (%)	15 (62,50%)		23 (63,89%)			
Low risk profile [n], (%)	2 (8,33%)		3 (8,33%)			
Adherence to SPC intervals (according to Sonnenschein et al.)[5]						
Fully adherent [n] (%)	4 (17.00%)		3 (8.00%)			
Partially adherent [n] (%)	4 (17.00%)		4 (11.00%)			
Insufficiently adherent [n] (%)	13 (54.00%)		23 (64.00%)			
Non-adherent [n] (%)	3 (13.00%)		6 (17.00%)			
Number of patients with extracted teeth during orthodontic treatment [n], (%)						
No teeth extracted [n], (%)	13 (54.17%)					
1 tooth [n], (%)	2 (8.33%)					
2 teeth [n], (%)	3 (12.50%)					
3 teeth [n], (%)	2 (8.33%)					
4 teeth [n], (%)	4 (16.67%)					
Orthodontic treatment need (according to Richmond et al.[41])						
Low [n], (%)	1 (4.17%)		3 (8.33%)			
Borderline [n], (%)	12 (50.00%)		21 (58.33%)			
High [n], (%)	11 (45.83%)		12 (33.33%)			

*POT* previous orthodontic treatment, *NOT* no orthodontic treatment, *T0* retrospective baseline examination ten years prior to T1, *T1* follow-up examination, *SD* standard-deviation, *min* minimum, *max* maximum, *SPC* supportive periodontal care. Other systemic diseases included osteoporosis, lung diseases, tumours in head/neck region, rheumatism, gout

### Orthodontic treatment

All patients of the POT-group were treated as adolescents with orthodontic appliances, with tooth extraction due to orthodontic reasons additionally performed in eleven patients (45.83%, range 1–4 teeth). Removable appliances were used in 17 patients (70.83%), fixed appliances in five patients (20.83%) and two patients (8.33%) received both fixed and removable appliances. Appliances were used in both jaws in 16 patients (66.67%), in seven patients (29.17%) only in the upper and in one patient (4.17%) only in the lower jaw. Duration of active OTx was in mean 2.8 ± 1.3 years (range: 2–7 years). Active OTx was completed on average 38.3 ± 8.8 years (range 21–59 years) prior to T0. No patient was under active OTx during the observation period. None of the patients received maxillofacial surgery.

### Clinical findings

At both time points, the NOT-group had a statistically significant higher CAL by approximately 0.5 mm (T0: *p* = 0.047; T1: *p* = 0.027). The PCR was significantly different between the groups at T0 (*p* = 0.001), but not at T1 (*p* = 0.076). Mean PPD in the NOT-group was 0.1 mm significantly higher than in the POT-group at T1 (*p* = 0.018). No other significant differences between the groups in terms of the clinical parameters were found (Table 2). At T0, one more patient in the POT group reached the clinical endpoint compared to the NOT group (*p* = 0.102). At T1, 19 patients in the POT group and 27 patients in the NOT group had reached the clinical endpoint (*p* = 0.950). Intra-group comparisons revealed a significant decrease in the number of teeth between T0 and T1 in both groups (POT: *p* = 0.004; NOT: *p* = 0.001). Additionally, a significant reduction in GBI (*p* = 0.0189) between T0 and T1 was observed in the NOT group. Overall, 12 additional patients in the NOT group reached the clinical endpoint between T0 and T1 (*p* = 0.0047). Between T0 and T1, 39 surgical procedures were performed on 26 patients.

**Table 2:** Clinical findings – comparison between groups

Parameter	T0			T1			∆ T0-T1		
	POT	NOT	p-value	POT	NOT	p-value	POT	NOT	p-value
Number of teeth [n], mean ±SD	26.08 ± 3.22	25.42 ± 2.96	*p=0.411* ^*u*^	25.17 ±3.37	24.78 ± 3.05	*p=0.538* ^*u*^	−0.92 ± 1.74 (p=0.004*^w^	−0.64 ± 0.90 (p=0.001*^w^)	*p=0.872* ^*u*^
PPD (mm), mean ±SD	2.34 ± 0.35	2.43 ± 0.45	*p=0.729* ^*u*^	2.31 ±0.24	2.48 ± 0.27	p=0.018* ^*u*^	−0.03 ± 0.33 *(p=0.648*^*w*^)	0.05 ± 0.51 *(p=0.338*^*w*^*)*	*p=0.298* ^*u*^
Rec (mm), mean ±SD	0.67 ± 0.73	1.13 ± 1.01	*p=0.120* ^*u*^	0.82 ±0.46	1.17 ± 0.87	*p=0.194* ^*u*^	0.15 ± 0.42 *(p=0.179*^*w*^*)*	0.04 ± 0.53 *(p=0.330*^*w*^*)*	*p=0.751* ^*u*^
CAL (mm), mean ±SD	3.02 ± 0.87	3.56 ± 1.09	p=0.047* ^*u*^	3.13 ±0.53	3.66 ± 0.88	p=0.027* ^*u*^	0.12 ± 0.59 *(p=0.424*^*w*^*)*	0.09 ± 0.66 *(p=0.371*^*w*^*)*	*p=0.809* ^*u*^
BOP (%), mean ±SD	10.75 ± 8.86	17.19 ± 14.83	*p=0.197* ^*u*^	13.96 ±9.47	17.72 ± 13.58	*p=0.201* ^*u*^	3.21 ± 7.99 *(p=0.059*^*w*^*)*	0.53 ± 15.76 *(p=0.494*^*w*^*)*	*p=0.827* ^*u*^
GBI (%), mean ±SD	1.79 ± 2.87	4.83 ± 8.51	*p=0.338* ^*u*^	1.46 ±2.47	1.53 ± 2.56	*p=0.946* ^*u*^	−0.33 ± 2.46 *(p=0.482*^*w*^*)*	−3.31 ± 7.33 (p=0.019*^w^*)*	*p=0.420* ^*u*^
PCR (%), mean ±SD	21.00 ± 16.50	31.14 ± 12.73	p=0.001* ^*u*^	26.75 ± 12.99	33.19 ± 13.57	*p=0.076* ^*u*^	5.75 ± 15.53 *(p=0.131*^*w*^*)*	2.06 ± 14.87 *(p=0.350*^*w*^*)*	*p=0.502* ^*u*^
Clinical endpoint reached^a^ [n], (%)	16 (66.67%)	15 (41.67%)	*p=0.102* ^x^	19 (79.17%)	27 (75.00%)	*p=0.950* ^*x*^	3 (12.50%) *(p=0.317*^*n*^*)*	12 (33.33%) (p=0.0047*^n^*)*	

*T0* retrospective baseline examination ten years (± six months) prior to T1, *T1* follow-up examination, *POT* previous orthodontic treatment, *NOT* noorthodontic treatment, *SD* standard deviation, *PPD* probing pocket depth, *Rec* recessions, *CAL* clinical attachment loss, *BOP* bleeding on probing, *GBI* gingival bleeding index, *PCR* plaque control record. *statistically significant difference, defined as p<0.05; ^u^Mann Whitney-U-test; ^w^Wilcoxonrank sum test; ^x^Chi-Square-test; ^n^McNemar’s test; ^a^according to Feres et al. [38].

### IOTN

Distribution of orthodontic treatment need at T1 is shown in Table 1. Spearman’s rank correlation coefficient indicated a negligible correlation between IOTN grade and the differences between T0-T1 for CAL, number of teeth, PPD, BOP, GBI, PCR, and TLP between T0 to T1 in all patients (Table 3) [43].

**Table 3:** Spearman’s rank correlation between the IOTN grade and difference in clinical parameters T0-T1

			*IOTN*	*Tooth loss* ^a^	*Number of teeth*	*PPD*	*Rec*	*CAL*	*BOP*	*GBI*	*PCR*
Spearman-Rho	IOTN	Coefficient	1,000	−0.051	0.196	0.017	−0.012	−0.026	−0.183	0.092	0.284

*IOTN* Index of Orthodontic Treatment Need [7], *PPD* probing pocket depth, *Rec* gingival recession, *CAL* clinical attachment loss, *BOP* bleeding on probing, *GBI* gingival bleeding index [26], *PCR* plaque control record27. ^a^Tooth loss due to periodontitis between T0 and T1

### Tooth loss and regression analysis for CAL-change

The multiple linear regression analysis for CAL change between T0 and T1 (Table 4) showed that, although not significant, the CAL change between T0 and T1 was on average 0.12 mm lower (95% CI: [−0.38; 0.13], *p* = 0.33) in the POT group compared to the NOT group. However, a significant effect of CAL at T0 (−0.44; 95% CI: [−0.57, −0.32], *p* < 0.001) was observed, indicating that patients with higher CAL values at T0 experienced, on average, a smaller change in CAL between T0 and T1. Moreover, the CAL change was significantly lower (−0.36 mm; 95%CI: [−0.61; −0.12], *p* = 0.004) for female compared to male patients. The other investigated parameters did not yield any statistically significant result. The variance inflation factor (VIF) indicates there is no multicollinearity issue with the variables included in the regression model. Assuming a sample size of 60 patients and an observed treatment group difference in mean CAL change between T0 and T1 of 0.22 ± 0.63 mm, the post-hoc power analysis yielded a 3.88% power to detect treatment group difference in CAL change when conducting a two-sided Student’s t-test at the 5% significance level. Assuming the same SD, a power of 80% would have been achieved if a group difference of 0.32 mm for the CAL difference between T0 and T1 would have been present. The assumptions of the multiple linear regression such as linearity, influential observations, homogeneity of variance, collinearity, normality of residuals were investigated graphically, showing no serious violations.

A total of 45 teeth were lost between T0 and T1, which corresponds to a mean loss of 0.75 teeth/patient over ten years (± six months). Ten patients lost in total 22 teeth over this period in the POT-group, with TLP limited to six teeth in three patients. In the NOT-group, 15 patients lost 23 teeth in total, of which ten teeth were TLP in seven participants. The negative binomial regression model indicates that the cumulative incidence of TLP for patients in the POT-group was 0.9 (95% CI: [0.21, 3.79]) times the incidence rate for the NOT-group. Nevertheless, this effect was not significant (*p* = 0.88, Table 4).

**Table 4:** Regression models

	*Estimate*	*95% CI*	*p-value*	*VIF*
(Intercept)	1.6	[0.26, 3]	0.021	
Group NOT POT	– −0.12	– [−0.38, 0.13]	– 0.33	1.1
CAL at T0 (in mm)	−0.44	[−0.57, −0.32]	< 0.001	1.2
Sex Male Female	– −0.36	– [−0.61, −0.12]	– 0.004	1.1
Age at T0 (in years)	0.01	[−0.01, 0.02]	0.24	1.2
Number of teeth at T0	−0.01	[−0.05, 0.03]	0.51	1
Smoking status at T0 Non-smoker Smoker	– 0.11	– [−0.23, 0.44]	– 0.53	1.2
	*Exp(B)*	*Lower 95% CI*	*p-value*	
(Intercept)	0.28	[0.11, 0.69]	0.005	
Group: POT	0.9	[0.21, 3.79]	0.88	

*n *60. *CAL* clinical attachment loss, *T0* retrospective baseline examination ten years (± six years) prior to T1, *T1* follow-up examination, *CI* Confidence Interval, *NOT *no orthodontic treatment, *POT* previous orthodontic treatment, *SPC* supportive periodontal care. Dispersion parameter for negative binomial distribution: 0.2657, *VIF* Variance Inflation Factor

## Discussion

Periodontitis patients with and without history of OTx did not exhibit a statistically significant difference between groups in the progression of CAL over ten years of SPC. Additionally, no correlation was found between the need for OTx according to the IOTN at follow-up and changes in periodontal and dental parameters throughout the observation period.

Bollen et al. found a correlation between the presence of malocclusion and periodontal disease and concluded, that the existing low-quality evidence suggests small detrimental effects to the periodontium through OTx [20]. Numerous studies found gingival recessions after OTx with increasing prevalence after a five-year observation period [44–48]. Macey et al. could not draw a clear conclusion on the effects of malocclusions and OTx on oral health based on the current state of studies [22]. Martin et al. found no statistically significant impact of orthodontic tooth movement on periodontal outcomes in non-periodontitis and stable treated periodontitis patients, based on a small number of low-quality studies [23]. This study did not find any possible risk factors of OTx for a deterioration in periodontal parameters, but several factors are discussed in the literature. Fixed buccal orthodontic appliances have been cited as possible influencing factors for worsening periodontal condition [49]. The effects of skeletal anchorage on the periodontium have not yet been sufficiently investigated due to the current state of research [23]. Regarding tooth movement, both intrusion and extrusion appear to be feasible in a reduced periodontium if periodontal inflammation can be adequately controlled [50]. In addition to oral hygiene, timing of the OTx and consideration of biomechanics, including force level and inclination level, should also be taken into account when performing OTx [51].

It is noticeable that patients without OTx had a significant higher CAL during the observation period compared to patients with a history of OTx, but the difference of 0.5 mm can be considered clinically not relevant. The results show that with regular SPC, periodontal conditions can be maintained over a long period of time even in patients with severe CAL. A clinically relevant lower PCR in the POT-group could be due to the fact that since beginning of active OTx, a focus was placed on sufficient oral hygiene. Ideally, this situation could be maintained, while patients in the NOT-group may have had to be trained during systematic periodontitis therapy. Since all patients were in SPC, both groups received regular oral hygiene instructions.

The observation of only minimal changes in PPD and CAL during long-term SPC is in accordance with other studies [52, 53]. With a mean TL rate of 0.075 per patient/year, the results of the present study are also in the range of those of other studies [54, 55]. Periodontitis stage and grade were comparable between T0 and T1 and the clinical endpoints according to Feres et al. [38] even improved in the NOT-group. Although the difference between groups was not significant, the fact that only 42% of the patients in the NOT-group reached the clinical endpoint at T0 may have an impact on the results of this study. It should be noted that the treatments were carried out in a specialised periodontology department of a university hospital, where severe cases and therapy-resistant forms of periodontitis regularly occur.

The approximately equal need for OTx between the groups seems surprising but was also observed in other studies [56]. Possible explanations could be an inadequate therapy due to a lack of adherence or unfavourable initial situations before OTx. In the POT-group, OTx might have reduced malocclusions and created a similar situation compared to untreated patients in the first place, which highlights the importance of standardised dental healthcare policies. In Germany, guidelines for statutory health insurances regarding OTx were adopted in 2003 [57], which might be a factor in the present study. In addition, there is a tendency for the teeth to return to their initial position without retention [58]. Only three patients had a removable or fixed retainer after active OTx, one of them was in situ at T1. While other studies have investigated the prevalence of malocclusions and their possible effect on periodontal health, this study examined a possible correlation between the current IOTN grade and the progression of periodontal parameters over long-term SPC. Taking into account the limitations, the results indicate that an increased IOTN does not correlate with the progression of periodontal and dental parameters during the last ten years of SPC. However, orthodontic treatment need as a potential risk factor for periodontal disease should be investigated in future prospective studies.

There are several limitations in this study. Since active OTx was carried out at an average of 38 years ago, it was not possible to evaluate whether the patients of the NOT-group did not need OTx, had unsuccessful OTx, refused OTx, or had higher IOTN before OTx. No socioeconomic factors were assessed to clarify, whether financial reasons prevented patients from undergoing OTx. However, the course of cost coverage for OTx in Germany over the last 20 years makes this seem unlikely [59]. Secondly, the IOTN, which was used in this study to assess orthodontic treatment need at T1, does not provide a clear assessment of the extent of orthodontic treatment required. Other indices might have provided a more differentiated picture of the patient’s orthodontic situation. OTx can vary greatly in terms of duration and severity. This leads to a high degree of heterogeneity among patients, not only in this study but throughout the existing literature. However, in order to provide a better overview of the orthodontic treatment carried out in this study, the information was presented descriptively. Effects of bias could have been caused by the patient acquisition process. Furthermore, included patients had received SPC for at least 9,5 years. Patients receiving less good care were therefore not considered in this study. A broader spectrum of patients and comparing the population with patients without APT would contribute to achieve a higher generalisability. To obtain results which can be compared to existing literature [23, 52], multiple measurements of periodontal parameters for each patient were aggregated in the analysis. Mainly periodontal parameters were investigated in this study. However, periodontitis also has an effect on tooth position. The importance of this context can be seen in the current classification of periodontitis: stage IV is defined by, among other things, masticatory dysfunction, secondary occlusal trauma, bite collapse, tooth migration and flare-ups [60]. Presence of malocclusions and periodontitis, the therapy and its effects should therefore always be considered together. The assessment of malocclusions could be an important variable for the possible progression of periodontal disease. Although this could not be carried out in this study due to its design, it offers great potential for future studies. Future work on this field should ideally have a prospective character with documentation of the initial situation, a larger sample size and further investigate other periodontal parameters in detail.

## Conclusion

Patients with and without a history of OTx showed a similar progression of periodontal parameters during ten years of SPC. In this population, patients without OTx had higher CAL at both time points than orthodontically treated patients, but both groups were able to maintain stable periodontal conditions through SPC. Patients in both groups had a similar need for OTx at follow-up, assessed by IOTN. No correlation between the need for OTx and the progression of periodontal and dental parameters within the previous ten years of SPC could be seen.

## Data Availability

Data of this study are available from the corresponding author upon reasonable request.

## Abbreviations

AC: Aesthetic component
APT: Active periodontitis therapy
BOP: Bleeding on probing
CAL: Clinical attachment loss
DHC: Dental health component
GBI: Gingival bleeding index
IOS: Intraoral scan
IOTN: Index of orthodontic treatment need
NOT: No previous orthodontic treatment
OTx: Orthodontic treatment
POT: Previous orthodontic treatment
PCR: Plaque control record
PPD: Probing pocket depth
SD: Standard deviation
SPC: Supportive periodontal care
TL: Tooth loss
TLP: Tooth loss due to periodontitis
T0: Visit T0 - baseline
T1: Visit T1 – follow-up
VIF: Variance inflation factor
3DM: Three-dimensional digital model
